# Potential of DPD ((S)-4,5-dihydroxy-2,3-pentanedione) Analogs in Microparticulate Formulation as Vaccine Adjuvants

**DOI:** 10.3390/ph17020184

**Published:** 2024-01-30

**Authors:** Devyani Joshi, Sarthak Shah, Christiane Chbib, Mohammad N. Uddin

**Affiliations:** 1 Center for Drug Delivery Research, Vaccine Nanotechnology Laboratory, College of Pharmacy, Mercer University, Atlanta, GA 30341, USA; devyani.jaideep.joshi@live.mercer.edu (D.J.); sarthak.modi.shah@live.mercer.edu (S.S.); 2 College of Pharmacy, Larkin University, 18301 N Miami Ave, Miami, FL 33169, USA; cchbib@larkin.edu

**Keywords:** adjuvant, (S)-DPD, microparticle, quorum sensing, PLGA

## Abstract

The molecule (S)-4,5-dihydroxy-2,3-pentanedione (DPD) is produced by many different species of bacteria and is involved in bacterial communication. DPD is the precursor of signal molecule autoinducer-2 (AI-2) and has high potential to be used as a vaccine adjuvant. Vaccine adjuvants are compounds that enhance the stability and immunogenicity of vaccine antigens, modulate efficacy, and increase the immune response to a particular antigen. Previously, the microparticulate form of (S)-DPD was found to have an adjuvant effect with the gonorrhea vaccine. In this study, we evaluated the immunogenicity and adjuvanticity of several synthetic analogs of the (S)-DPD molecule, including ent—DPD((R)-4,5-dihydroxy-2,3-pentanedione), n-butyl—DPD ((S)-1,2-dihydroxy-3,4-octanedione), isobutyl—DPD ((S)-1,2-dihydroxy-6-methyl-3,4-heptanedione), n-hexyl—DPD ((S)-1,2-dihydroxy-3,4-decanedione), and phenyl—DPD ((S)-3,4-dihydroxy-1-phenyl-1,2-butanedione), in microparticulate formulations. The microparticulate formulations of all analogs of (S)-DPD were found to be noncytotoxic toward dendritic cells. Among these analogs, ent—DPD, n-butyl—DPD, and isobutyl—DPD were found to be immunogenic toward antigens and showed adjuvant efficacy with microparticulate gonorrhea vaccines. It was observed that n-hexyl—DPD and phenyl—DPD did not show any adjuvant effect. This study shows that synthetic analogs of (S)-DPD molecules are capable of eliciting adjuvant effects with vaccines. A future in vivo evaluation will further confirm that these analogs are promising vaccine adjuvants.

## 1. Introduction

Vaccination is used to generate a strong immune response against a particular pathogen, and this response may provide long-term protection against an infection. Adjuvants are compounds added to vaccine formulations to enhance the response against co-inoculated antigens. The word adjuvant originates from the Latin word adjuvare, which means ‘to help or to enhance’ [1,2,3]. Functionally, adjuvants increase the immunogenicity of antigens, speed up the mechanism, enhance the duration of the immune response, stimulate cell-mediated immunity, promote the induction of mucosal immunity, amplify the immune response in immunologically immature individuals, and reduce the dose of antigen necessary, lowering vaccine costs [4,5]. Traditional vaccines use weakened or inactivated pathogens as antigens that are heterogeneous and contain many epitopes. Some of these epitopes provide an extended T-cell response, but their contribution to immunogenicity is minuscule (6). For instance, mRNA vaccines offer more benefits than traditional vaccines such as inexpensive production, easy scale-up, extended safety, and high-level antigen expression. There are still several unanswered questions about mRNA vaccines including challenges associated with the vaccines’ development and use [6]. There are several types of current vaccines, and their advantages and limitations are shown in Table 1 [7,8]. Therefore, there is a dire need for an appropriate immunological adjuvant that are potent, safe, and compatible with new generations of vaccines [9].

Quorum sensing (QS) is a bacterial communication process that allows bacteria to regulate their gene expression and act together as a population [10,11,12,13,14]. QS molecules are able to trigger the biological process during this communication process that regulates several pathogenic processes including virulence factor production, antibiotic susceptibility, and biofilm formation. Thus, QS modulation can serve as a potential curative or preventive approach to fight bacterial infections [15,16,17]. The QS communication process is mediated by the release and response to small molecules known as autoinducers (AI). Autoinducer-2 (AI-2) regulates intra- and interspecies bacterial communication that is capable of eliciting responses in different species of bacteria and is known as the “universal autoinducer”. However, targeting AI-2-based QS molecules is challenging due to the fact that they exist in both linear and cyclic forms and undergo rapid interconversion from the cyclic to the linear form and vice versa. The linear and cyclic forms are recognized by different bacteria [18,19,20,21]. There are several systems that are involved in QS. Specifically, this includes the AHL system, AIP (or AI-1 system previously), and AI-2 system. The AHL system primarily works in Gram-negative bacteria and the signaling molecules are N-acyl homoserine lactones (AHLs). In the AI-1 QS, this system focuses on Gram-positive bacteria. AI-2 signaling molecules are a class of furanosyl borate diesters for which the precursors are 4,5-dihydroxy-2,3-glutaradione (DPD). The DPD molecule is common between Gram-positive and Gram-negative bacteria. Previously, DPD or autoinducer-2 was determined as immunogenic and had adjuvant potential in our previous paper. The DPD molecule, when converted into a microparticle form, acted as an adjuvant when paired with several antigens [22].

Regarding the quorum sensing bacterial communication process in the small molecule Autoinducer I (AI), the gram-negative and gram-positive bacteria show different pathways of mechanism of action in the quorum sensing process. How bacteria generally communicate in the quorum sensing process is based on a few general steps. Generally, the QS communication process depends on three basic rules. Initially, the bacteria involved in communication produce AIs, which are the signaling molecules. At a low cell density (LCD) of the bacterial population, AIs diffuse away, which is present at lower concentrations. On the other hand, at a high cell density (HCD), the production of AIs increases and results in a high concentration that causes the initiation of detection and response. Secondly, at high concentrations, the AIs are detected by receptors that exist in the cytoplasm or in the membrane. Finally, the detection of AI by neighboring bacteria becomes activated and produces its own AI to further increase the communication process [21].

**Figure 1:**
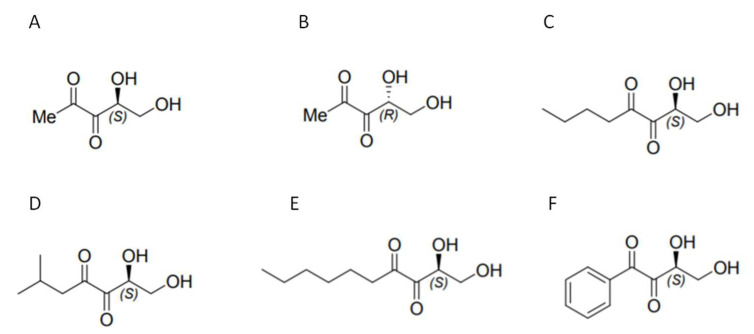
Structures of DPD and its analogs. (**A**) DPD ((S)-4,5-dihydroxy-2,3-pentanedione); (**B**) ent—DPD((R)-4,5-dihydroxy-2,3-pentanedione); (**C**) n-butyl—DPD ((S)-1,2-dihydroxy-3,4-octanedione); (**D**) isobutyl—DPD ((S)-1,2-dihydroxy-6-methyl-3,4-heptanedione); (**E**) n-hexyl—DPD ((S)-1,2-dihydroxy-3,4-decanedione); (**F**) phenyl—DPD ((S)-3,4-dihydroxy-1-phenyl-1,2-butanedione).

In previous studies conducted in our lab, we evaluated the potential of the (S)-DPD molecule in microparticulate form as a possible vaccine adjuvant. We found that microparticulate (S)-DPD acted as an adjuvant with a microparticulate gonorrhea vaccine [23]. Therefore, in this study, as a continuation of our previous finding, we investigated the potential of synthetic analogs of (S)-DPD as probable vaccine adjuvants. The synthetic analog molecules of (S)-DPD that have been tested are described in Figure 1.

**Figure 2:**
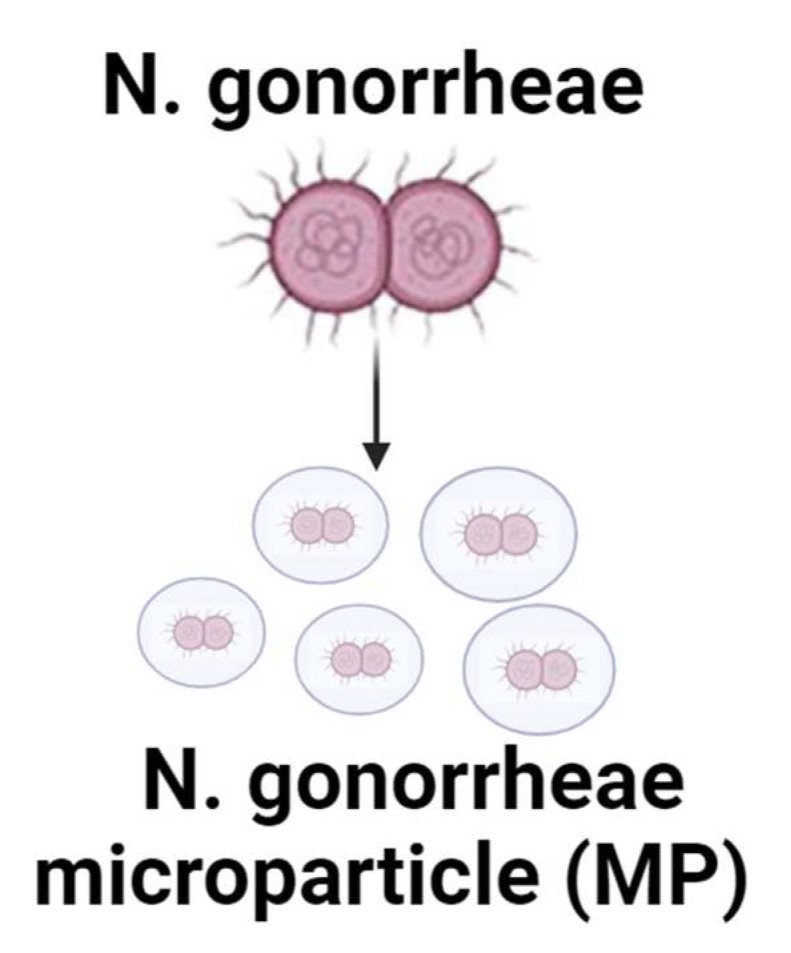
The whole-cell inactivated *N. gonorrhea* was encapsulated into a polymer matrix to form vaccine microparticles (MP).

The development of an effective gonorrhea vaccine has been demanding because of the antigenic variability of *N. gonorrhea* and its ability to inhibit the development of adaptive immune responses. Previous endeavors to formulate an effective gonorrhea vaccine, either whole-cell attenuated or subunit recombinant, were unsuccessful. The clinical representation in symptomatic males versus female patients is different. In males, symptoms include urethral discharge, dysuria, and testicular or different levels of rectal pain. In females, symptoms include vaginal discharge, dysuria, dyspareunia, abnormal uterine bleeding, and lower abdominal and/or rectal pain. In terms of the pathological aspect, N. gonorrhoeae has the ability to colonize the genital mucosa. However, this infection can also colonize other mucosa such as ocular, nasopharyngeal, and anal mucosa. Once the immune system is activated, there can be further complications from untreated, ascending, genital-tract infections. In women, this infection can lead to pelvic inflammatory disease, infertility, and ectopic pregnancy [24,25]. In this study, we used a gonorrhea vaccine that was previously formulated in our laboratory, in which whole-cell inactivated *N. gonorrhea* was entrapped in a polymer matrix of a microparticle formulation [26,27]. The *N. gonorrhea* microparticle (MP) is shown in Figure 2.

Depending on their function, adjuvants can be classified as immunostimulatory adjuvants or vaccine delivery systems [3,28,29]. Immunostimulatory adjuvants include mineral salts, immunostimulatory complexes, polysaccharides, and microbial-derived products [30]. Vaccine delivery systems include liposomes, and micro- and nanoparticles [31]. There are currently six types of FDA-approved adjuvants on the market: aluminum salts, monophosphoryl lipid A, oil-in-water emulsion, CpG, and QS-21 saponin [32,33]. Aluminum adjuvants are the most common adjuvants among the marketed products. Aluminum adjuvants elicit several adverse effects that are related to their mechanism of action. Such effects include aluminum-induced injection site pain and tenderness [34], which may reflect cell necrosis and the induction of inflammasome activation and IL-1 production [35].

Particulate adjuvants have particle sizes comparable to those of pathogens. Generally, smaller particles (<5 µm) are more immunogenic than larger particles. Particulate adjuvants can also modulate the type of immune response toward the co-inoculated antigen and aid in cross-presentation of the antigen, which is a prime role for generating CD8^+^ T-cell responses. Moreover, adding up such potent immunostimulatory adjuvants into delivery systems may reduce adverse events by limiting the systemic circulation of the adjuvant [36,37,38]. By formulating the various analogs of (S)-DPD into microparticles, we are consolidating the advantages of both classes of adjuvants.

The microparticles were formulated using the biocompatible and biodegradable polymer PLGS (poly (lactic-co-glycolic acid)) matrix. PLGA particles are known to increase the potency of vaccine and adjuvant formulations. Previously, several investigations have indicated the significance of PLGA particulate vaccines in stimulating robust Th1-type immune responses that are detected by the secretion of IgG2a antibodies. Moreover, these adjuvant-containing particles enhance and prolong the cross-presentation efficiency [39,40,41].

In this study, we evaluated the adjuvant effect of microparticulate formulations loaded with various (S)-DPD analogs. We initially tested these microparticulate formulations of analogs for their cytotoxicity and immunogenicity profiles, which was followed by evaluating their adjuvant potential upon combination with the microparticulate gonorrhea vaccine that was previously developed in our laboratory. We compared the adjuvant potential of these microparticulate formulations with the adjuvant effect of FDA-approved adjuvants as microparticulate formulations.

## 2. Results

### 2.1. Microparticle Formulation and Quatitation

The percentage of yield of the microparticulate formulations of various DPD analogs ranged from 86 to 92% (Table 2). The loss of particles can be attributed to material loss during homogenization and centrifugation. The numbers in Table 2 represent the parameters of percentage of yield (%), size of particles (nm), polydispersity index (PDI), and surface zeta potential (mV) of particles. The recovery yield is measured to evaluate the percent of product. In this study, all the DPD analogs showed a higher recovery yield. The particle size is important for antigen uptake by antigen-presenting cells (APCs). The polydispersity index (PDI) is an indicator of the uniform size distribution of the particles. In Table 1, for all analogs, the results showed a lower PDI which indicates that our microparticles were of uniform size distribution. We also measured the zeta or charge of the MPs. The charges for all the DPD analogs were negative. The negative surface charge indicates the elevated stability of microparticles in aqueous solution.

### 2.2. Particle Size Analysis

A Malvern Nano Zetasizer (ZS) was used to measure the particle size of various microparticulate formulations loaded with different analogs of (S)-DPD. The particle size ranged from 350 to 500 nm (Table 1). We observed no significant differences between the size of microparticulate formulations and blank (unloaded) microparticles (n = 6).

### 2.3. Zeta Potential Measurement

The surface zeta potential value of the DPD analog-loaded microparticles was determined by using a Malvern Nano ZS and ranged from −30 to −35 mV (Table 1). No significant difference between the zeta potential of the blank (unloaded) microparticles and DPD analog-loaded microparticles was observed.

### 2.4. Scanning Electron Microscopy (SEM) Analysis

**Figure 3:**
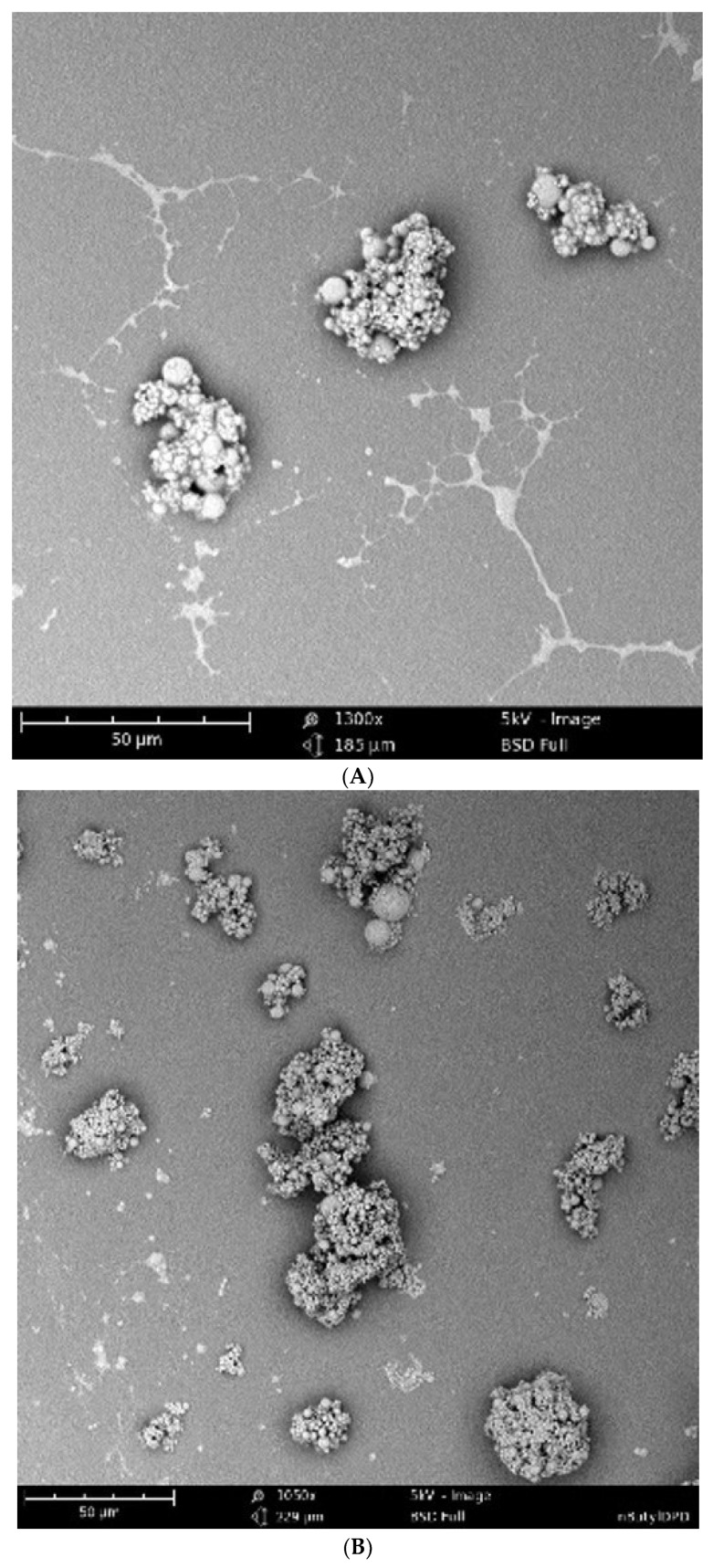

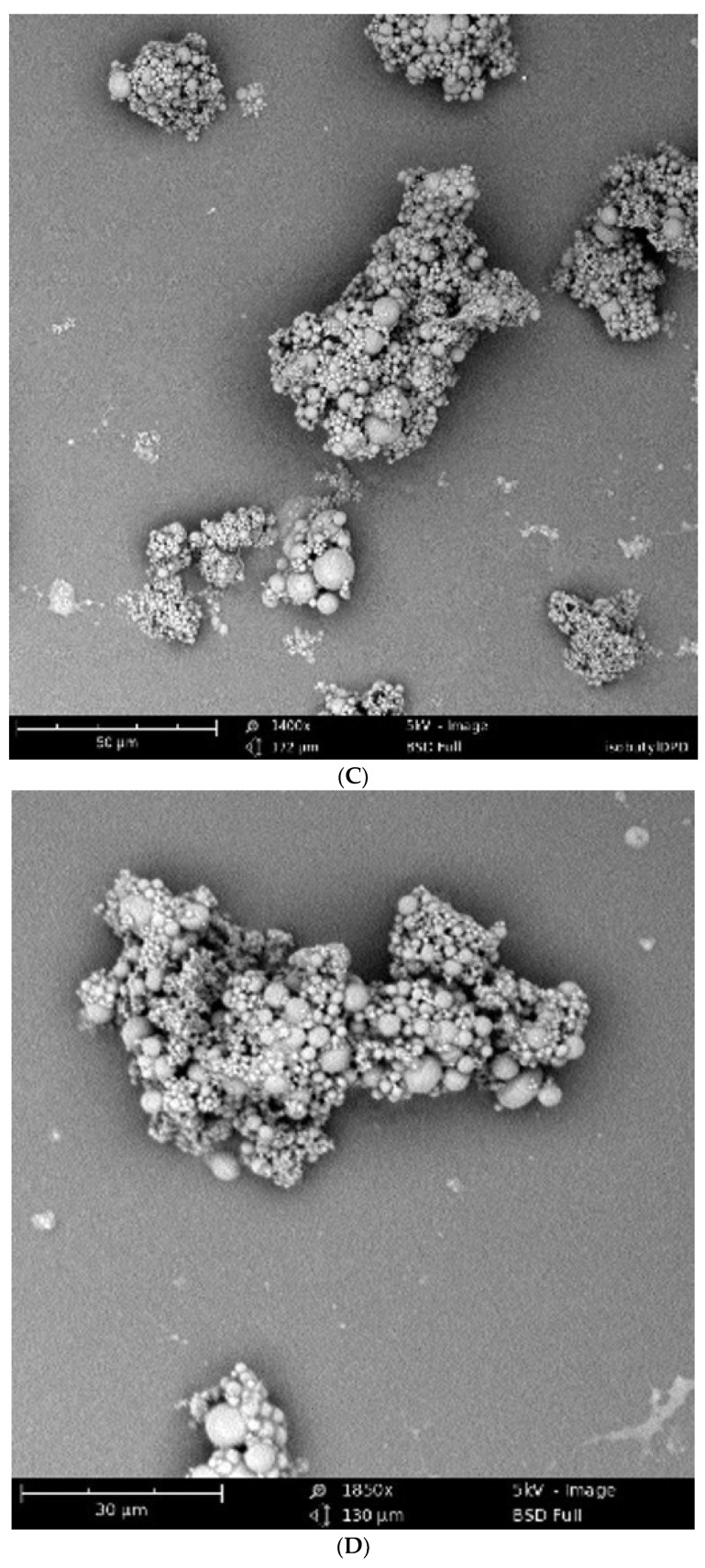

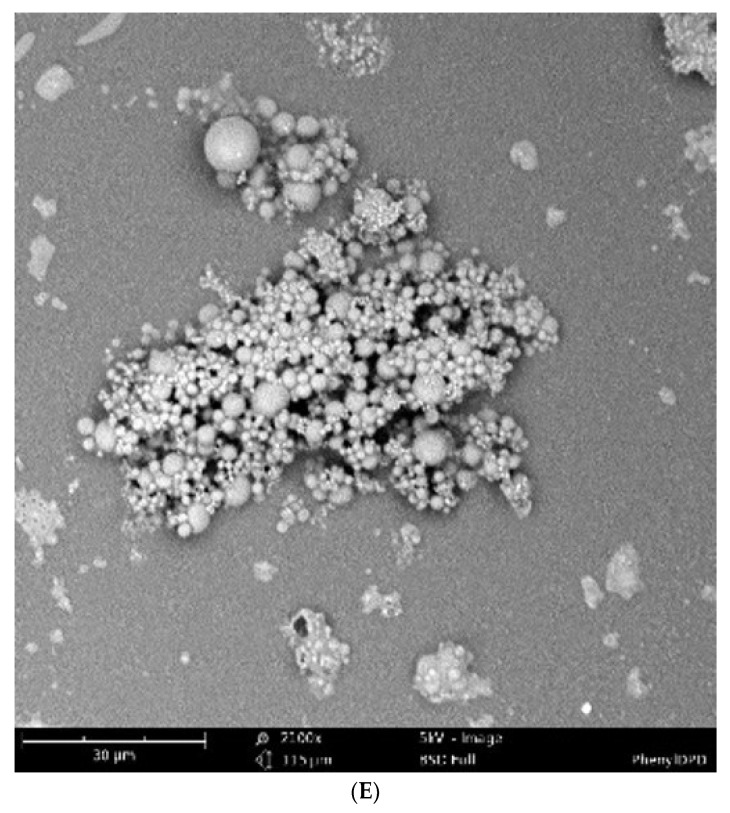
SEM (scanning electron microscopy) images of microparticulate DPD analogs. (**A**) ent—DPD, (**B**) n-butyl—DPD, (**C**) isobutyl—DPD, (**D**) n-hexyl—DPD, (**E**) phenyl—DPD. Scale bar: 30–50 µm.

In scanning electron microscopy (SEM), the surface morphology of the DPD analog-loaded microparticles was pictured (Figure 3). The shape of the microparticles was found to be spherical with no deformities. The SEM figures show that the particles were aggregated. We assume that the colloidal stability of the biodegradable PLGA strongly affected in aqueous solution which may have resulted in the aggregation of particles [42].

### 2.5. Griess’s Assay Analysis

**Figure 4:**
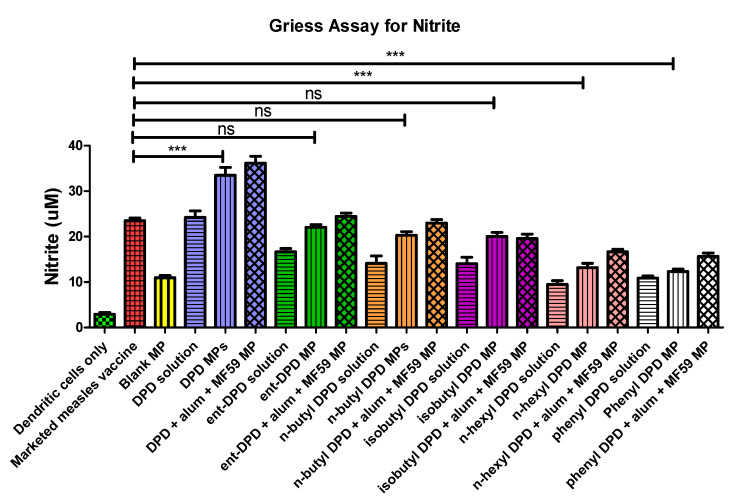
Level of nitric oxide released from dendritic cells was measured in Griess’s assay. Murine dendritic cells (DC2.4) (5 × 10^5^ cells) were pulsed with different groups of microparticles (dose 50 µg/5 × 10^5^ cells) for 48 h. Nitrite level in supernatant was measured using Griess’s reagents. There was no significant difference between nitric oxide released from marketed measles vaccine compared to ent—DPDMPs, n-butyl—DPD MPs, and isobutyl—DPD MPs. However, cells exposed to marketed measles vaccine released remarkably higher amounts of nitric oxide than the groups of cells exposed to n-hexyl—DPD MPs and phenyl—DPD MPs. Results are presented as the mean ± SEM (n = 3). *** *p* < 0.001 extremely significant.

Nitric oxide release is an important marker used to assess the induction of the innate immune response. Increased nitric oxide release indicates an increased immune response induction. In this study, nitric oxide release from exposed dendritic cells was set up as a positive control group, and only dendritic cells were used as the negative control group. The nitric oxide release from blank microspheres, the DPD solution, and DPD and its analogs with adjuvant alum and MF59 MP was also determined. The nitric oxide release from dendritic cells exposed to the positive control group (marketed measles vaccine) was comparable to that from the dendritic cells exposed to microparticulate ent—DPD, n-butyl—DPD, and isobutyl—DPD. However, the cells exposed to n-hexyl—DPD and phenyl—DPD microparticles released significantly lower amounts of nitric oxide when compared to those exposed to the positive control (Figure 4). This indicates that the microparticulate formulations of ent—DPD, n-butyl—DPD, and isobutyl—DPD had an immunogenicity equivalent to that of the marketed measles vaccine, while the immunogenicity of n-hexyl—DPD and phenyl—DPD microparticles was lower.

### 2.6. Cytotoxicity Study

**Figure 5:**
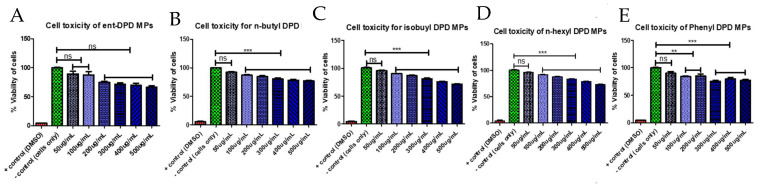
DPD analogs microparticles ((**A**) ent—DPD MPs, (**B**) n—butyl-DPD MPs, (**C**) isobutyl—DPD MPs, (**D**) n-hexyl—DPD MPs, and (**E**) phenyl—DPD MPs) were found to be noncytotoxic in dendritic cells (DCs). DCs were pulsed with increasing doses of microparticulate DPD analogs and incubated for 48 h. The microparticles were not cytotoxic compared to dimethyl sulfoxide (DMSO). Here, we compared the cells only group versus the lowest concertation, which was 50 ug/mL. This indicates that the lowest concentration is safe to use as a dose using DCs. In addition, the cells were only used to compare them versus other concentrations. Cytotoxicity of each analog was analyzed MTT (3-(4,5-dimethylthiazol-2-yl)-2,5-diphenyl tetrazolium bromide) reagent, which uses the reducing power of living cells to measure percentage of live cells. Data are expressed as the mean ± SEM (n = 3). ** *p* < 0.01 very significant, *** *p* < 0.001 extremely significant.

The microparticulate DPD analogs (ent—DPD, n-butyl—DPD, isobutyl—DPD, n-hexyl—DPD, and phenyl—DPD) were noncytotoxic toward dendritic cells at all tested concentrations (50 µg/mL to 500 µg/mL) (Figure 5).

### 2.7. In Vitro Release Study of Analogs of DPD

**Figure 6:**
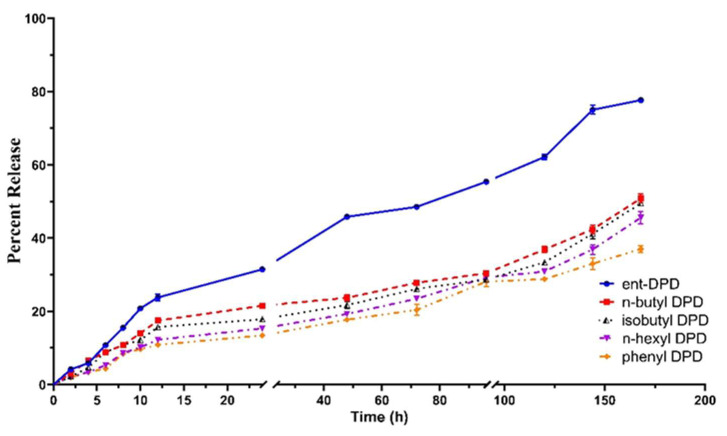
Release profile of DPD analogs from the PLGA polymer in phosphate buffer saline (PBS) at pH 7.4 and 37 °C. Analogs of DPD: ent—DPD ((R)-4,5-dihydroxy-2,3-pentanedione), n-butyl—DPD ((S)-1,2-dihydroxy-3,4-octanedione), isobutyl—DPD ((S)-1,2-dihydroxy-6-methyl-3,4-heptanedione), n-hexyl—DPD ((S)-1,2-dihydroxy-3,4-decanedione), and phenyl—DPD ((S)-3,4-dihydroxy-1-phenyl-1,2-butanedione). The study was performed for 168 h. Results are expressed as mean ± SEM (n = 6). A 50% release of ent—DPD was observed from the PLGA polymer at 48 h. All other analogs displayed a slower release profile than ent—DPD (50% release at approximately 168 h).

A release study of synthetic analogs of (S)-DPD, including ent—DPD((R)-4,5-dihydroxy-2,3-pentanedione), n-butyl—DPD ((S)-1,2-dihydroxy-3,4-octanedione), isobutyl—DPD ((S)-1,2-dihydroxy-6-methyl-3,4-heptanedione), n-hexyl—DPD ((S)-1,2-dihydroxy-3,4-decanedione), and phenyl—DPD ((S)-3,4-dihydroxy-1-phenyl-1,2-butanedione), was performed to evaluate the percentage of DPD analog molecules that are released from the PLGA polymer matrix for up to 168 h (7 days). A 50% release of ent—DPD from the PLGA polymer was observed at 48 h. The other analogs, n-butyl—DPD, isobutyl—DPD, n-hexyl—DPD, and phenyl—DPD, displayed a much slower release profile from the polymer. The analogs n-butyl—DPD and isobutyl PD exhibited a 50% release profile at approximately 168 h. The combined release profiles or all analogs are shown in Figure 6.

### 2.8. Expression of Antigen-Presenting Molecules

**Figure 7:**
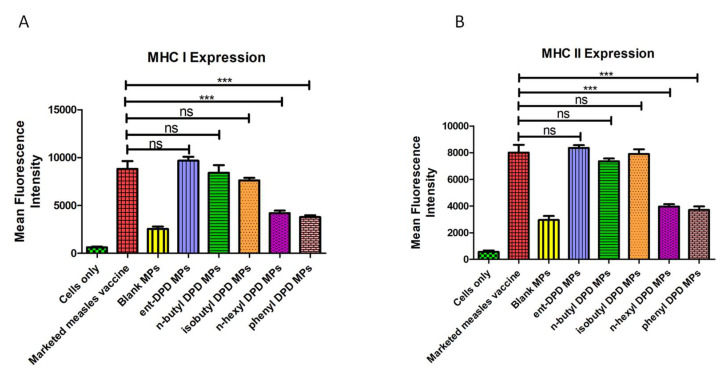
Major histocompatibility complexes I and II. (**A**) MHC I expression; (**B**) MHC II expression on the surface of murine dendritic cells (DC 2.4). Results are expressed as mean ± SEM (n = 3). *** *p* < 0.001 extremely significant.

In the in vitro study, it was detected that the dendritic cells exposed to ent—DPD, n-butyl—DPD, and isobutyl—DPD microparticles showed a comparable expression of the MHC I and MHC II antigen-presenting molecules as the cells exposed to the marketed measles vaccine (positive control). However, the dendritic cells showed significantly lower expression upon exposure to the n-hexyl—DPD microparticles and phenyl—DPD microparticles compared to the positive control. This showed that the immunogenicity of ent—DPD, n-butyl—DPD, and isobutyl—DPD microparticles was comparable to that of the marketed measles vaccine, while n-hexyl—DPD microparticles and phenyl—DPD microparticles were significantly less immunogenic than the vaccine (Figure 7).

### 2.9. Expression of Costimulatory Molecules

**Figure 8:**
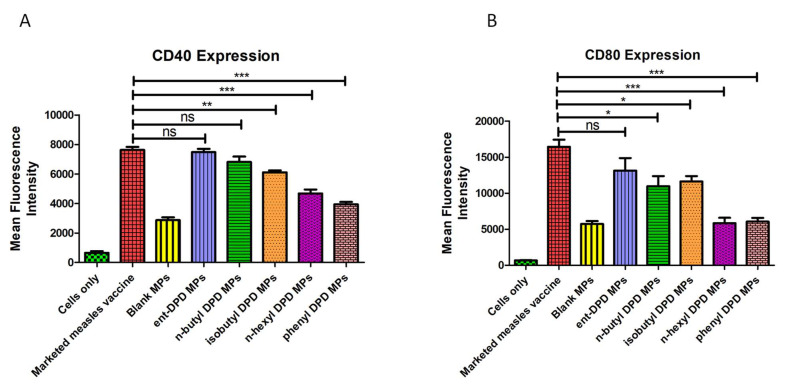
Costimulatory molecules CD40 and CD80. Level of (**A**) CD40 and (**B**) CD80 on the surface of murine dendritic cells. Results are expressed as mean ± SEM (n = 3). Dendritic cells showed significantly lower expression upon exposure to the n-hexyl—DPD microparticles and phenyl—DPD microparticles compared to the positive control. The term “There was no significant difference between” was used to compare the different groups based on the results of the statistical test. In Figure 7, for instance, there was no significant difference between the marketed measles vaccine versus the ent—DPDMPs and n-butyl—DPD MPs. This indicates that even though non-significant, ent—DPDMPs and n-butyl—DPD MPs showed similar immunostimulatory potential as the positive control, which was a marketed vaccine. In this manner, the DPD analogs ent—DPD and n-butyl—DPD worked similarly to the marketed vaccine. * *p* < 0.05, ** *p* < 0.01, *** *p* < 0.001 extremely significant.

There was no significant difference in terms of the expression of the costimulatory molecule CD40 between the cells that were exposed to the marketed measles vaccine and ent—DPD and n-butyl—DPD microparticles. However, the cells exposed to isobutyl—DPD, n-hexyl—DPD, and phenyl—DPD microparticles showed a significantly lower expression of CD40 than the cells exposed to the marketed vaccines. This indicates that the ent—DPD and n-butyl—DPD microparticles had an immunogenicity comparable to that of the marketed measles vaccine. However, there was a significant difference in the immunogenicity between the marketed measles vaccine and analogs of isobutyl—DPD, n-hexyl—DPD, and phenyl—DPD microparticles (Figure 8A).

For the expression of the costimulatory molecule CD80, there was no significant difference between the cells exposed to the marketed measles vaccine and those exposed to the ent—DPD microparticles. However, the cells exposed to n-butyl—DPD, isobutyl—DPD, n-hexyl—DPD, and phenyl—DPD microparticles showed significantly lower expression of the costimulatory molecule CD80 than the cells exposed to the marketed measles vaccine. This indicates that the DPD microparticles have an immunogenicity comparable to that of the marketed measles vaccine. However, the immunogenicity of n-butyl—DPD, isobutyl—DPD, n-hexyl—DPD, and phenyl—DPD microparticles was significantly lower than that of the marketed measles vaccine (Figure 8B).

### 2.10. Evaluation of Adjuvant Effect: Griess’s Assay for Nitrite

**Figure 9:**
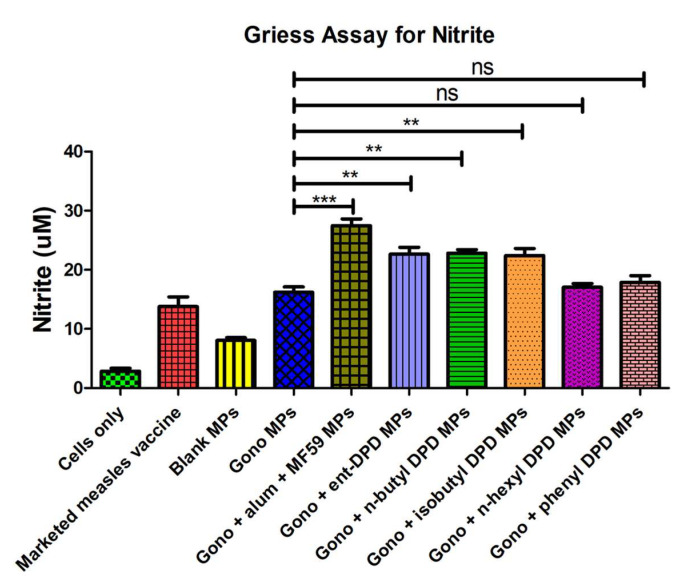
Release of nitric oxide from dendritic cells as calculated from Griess’s assay. Results are expressed as mean ± SEM (n = 3). ** *p* < 0.01 very significant; *** *p* < 0.001 extremely significant.

Nitric oxide release from antigen-presenting cells (APCs) is a hallmark of the induction of the innate immune response (32). In this study, it was found that when the dendritic cells were faced with gonorrhea vaccine microparticles combined with ent—DPD, n-butyl—DPD, and isobutyl—DPD microparticles, they released remarkably higher amounts of nitric oxide than the cells faced with gonorrhea vaccine microparticles only. However, there were no notable differences observed in terms of nitric oxide released from the cells faced with the combination of gonorrhea vaccine microparticles, n-hexyl—DPD microparticles, and phenyl—DPD microparticles in comparison with the cells that faced only gonorrhea vaccine microparticles. (Figure 9). Thus, ent—DPD, n-butyl—DPD, and isobutyl—DPD microparticles show an adjuvant effect with gonorrhea vaccine microparticles.

### 2.11. Evaluation of Adjuvant Effect with Bacterial and Viral Vaccines: Griess’s Assay for Nitrite

**Figure 10:**
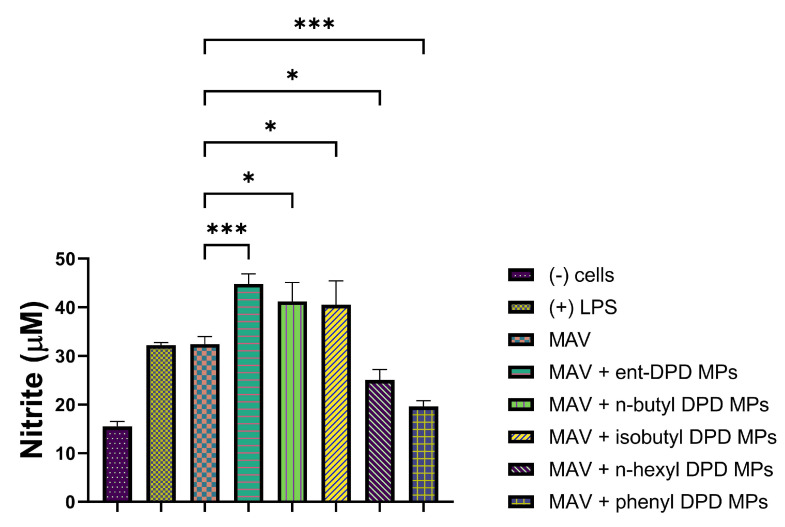
MenAfriVac (MAV), a meningococcal A conjugate vaccine, was combined with ent—DPD, n-butyl—DPD, isobutyl—DPD MPs, n-hexyl—DPD MPs, and phenyl—DPD MPs. Release of nitric oxide from dendritic cells as calculated from Griess’s assay. Results are expressed as mean ± SEM (n = 3). * *p* < 0.05 significant; *** *p* < 0.001 extremely significant.

**Figure 11:**
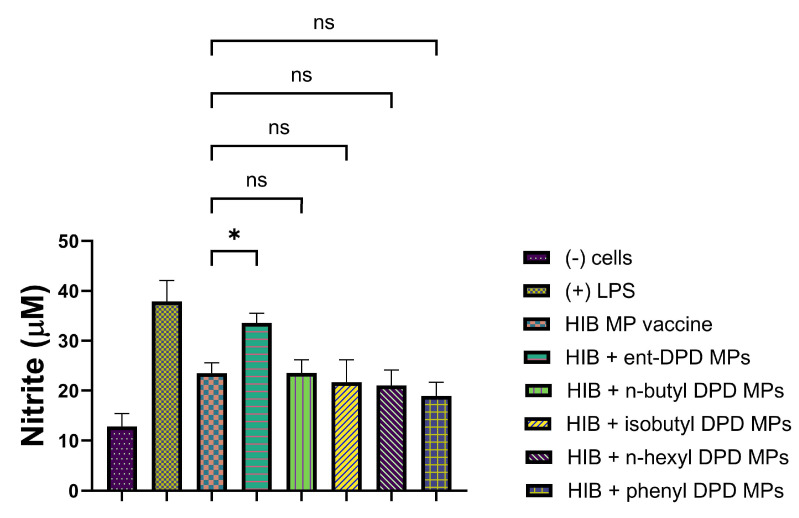
Hemophilus influenzae type B (HIB) conjugate vaccine microparticles were combined with ent—DPD, n-butyl—DPD, isobutyl—DPD MPs, n-hexyl—DPD MPs, and phenyl—DPD MPs. Release of nitric oxide from dendritic cells as calculated from Griess’s assay. Results are expressed as mean ± SEM (n = 3). * *p* < 0.05 significant.

**Figure 12:**
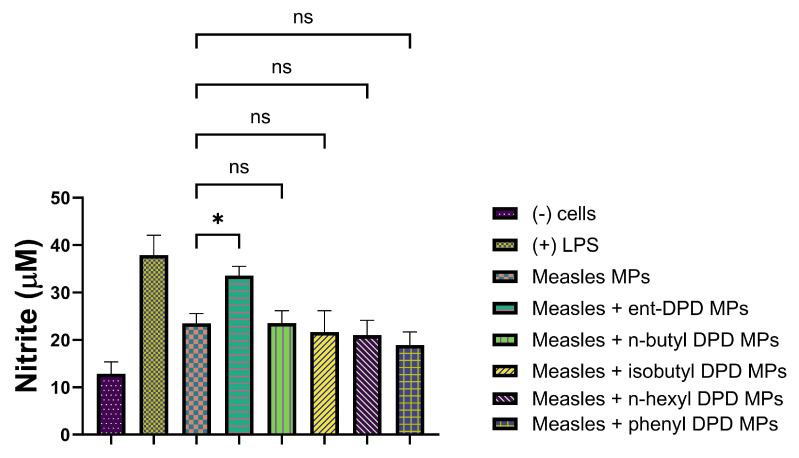
Measles vaccine microparticles were combined with ent—DPD, n-butyl—DPD, isobutyl—DPD MPs, n-hexyl—DPD MPs, and phenyl—DPD MPs. Release of nitric oxide from dendritic cells as calculated from Griess’s assay. Results are expressed as mean ± SEM (n = 3). * *p* < 0.05 significant.

**Figure 13:**
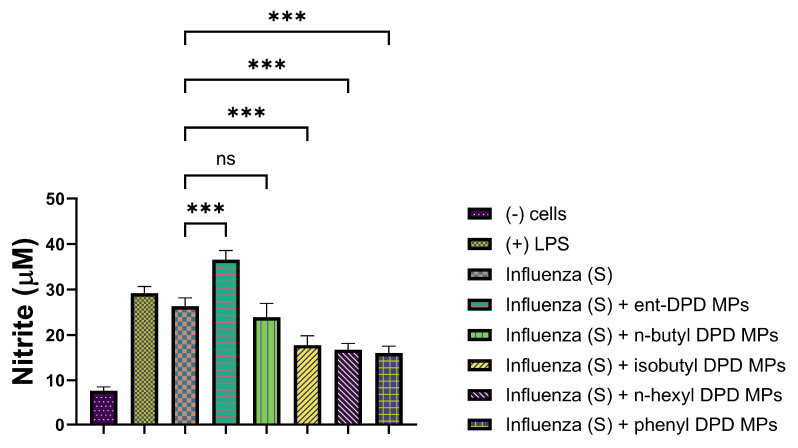
Influenza (S) vaccine was combined with ent—DPD, n-butyl—DPD, isobutyl—DPD MPs, n-hexyl—DPD MPs, and phenyl—DPD MPs. Release of nitric oxide from dendritic cells as calculated from Griess’s assay. Results are expressed as mean ± SEM (n = 3). *** *p* < 0.001 extremely significant.

**Figure 14:**
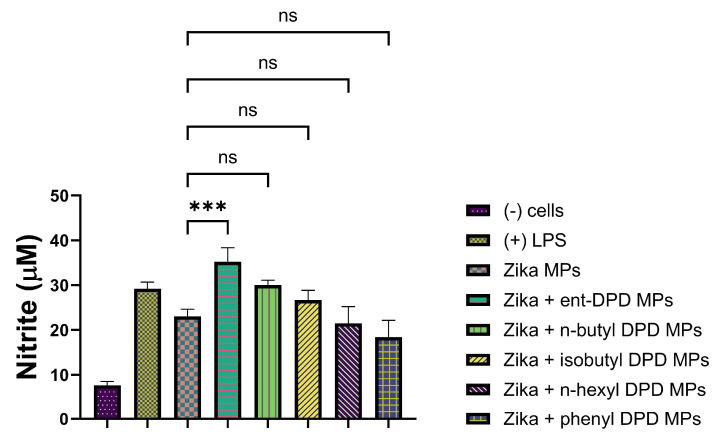
Zika vaccine microparticles were combined with ent—DPD, n-butyl—DPD, isobutyl—DPD MPs, n-hexyl—DPD MPs, and phenyl—DPD MPs. Release of nitric oxide from dendritic cells as calculated from Griess’s assay. Results are expressed as mean ± SEM (n = 3). *** *p* < 0.001 extremely significant.

We further tested the MP adjuvants with the analogs of ent—DPD, n-butyl—DPD, isobutyl—DPD, n-hexyl—DPD, and phenyl—DPD for their adjuvant potential by combining them with several bacterial and viral vaccines. We evaluated the adjuvant potential using two bacterial vaccines: MenAfriVac (MAV), a meningococcal A conjugate microparticle (Figure 10), and the Hemophilus influenzae type B (HIB) conjugate microparticle (Figure 11). We also evaluated the adjuvant potential with the following viral vaccines: measles vaccine microparticles (Figure 12), influenza (solution) vaccine (Figure 13), and inactivated Zika microparticles (Figure 14). Three adjuvants, ent—DPD, n-butyl—DPD, and isobutyl—DPD, showed adjuvant potential when combined with the MAV MP vaccine. When the compounds were tested with the HIB MP vaccine, measles MP vaccine, and Zika MP vaccine, only ent—DPD produced significant differences from the vaccine alone. When the MP adjuvants were combined with the influenza (S) vaccine, ent—DPD produced a significant response versus the vaccine alone. In addition, the influenza (S) vaccine and n-butyl MP produced similar responses. The results are shown in the following figures.

#### 2.11.1. Evaluation of Adjuvant Effect with MenAfriVac (MAV), a Meningococcal A Conjugate Vaccine Microparticle

The following figure shows the adjuvant effect of MenAfriVac (MAV), Meningococcal A Conjugate Vaccine Microparticle with all five types of DPD Analogs. 

#### 2.11.2. Evaluation of Adjuvant Effect with Hemophilus Influenzae Type B (HIB) Conjugate Vaccine Microparticles

The following figure shows the adjuvant effect of Hemophilus Influenzae Type B (HIB) Conjugate Vaccine Microparticles combined with all five types of DPD Analogs. 

#### 2.11.3. Evaluation of Adjuvant Effect with Measles Vaccine Microparticles

The following figure shows the adjuvant effect of Measles Vaccine Microparticles combined with all five types of DPD Analogs.

#### 2.11.4. Evaluation of Adjuvant Effect with Influenza (S) Vaccine

The following figure shows the adjuvant effect of Influenza Vaccine combined with all five types of DPD Analogs.

#### 2.11.5. Evaluation of Adjuvant Effect with Inactivated Zika Vaccine Microparticles

The following figure shows the adjuvant effect of Zika Vaccine combined with all five types of DPD Analogs.

### 2.12. Evaluation of Adjuvant Effect: Expression of Antigen-Presenting Molecules

**Figure 15:**
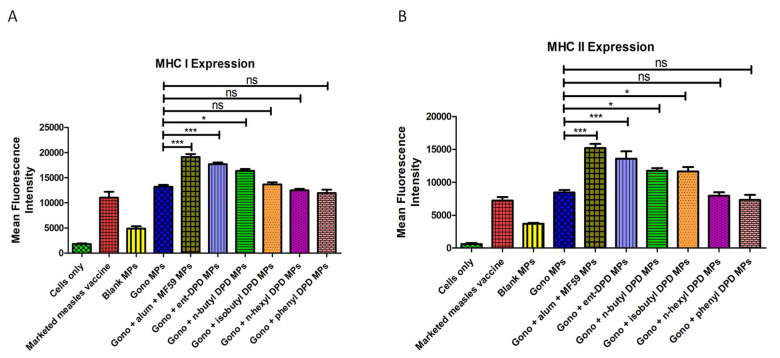
(**A**) MHC I expression; (**B**) MHC II expression on the surface of murine dendritic cells (DC 2.4). Results are expressed as the mean ± SEM (n = 3). * *p* < 0.05 significant; *** *p* < 0.001 extremely significant.

Dendritic cells exposed to the combination of the microparticulate gonorrhea vaccine and ent—DPD microparticles and n-butyl—DPD microparticles displayed a notably higher level of the antigen-presenting MHC I molecule than those exposed to only gonorrhea vaccine microparticles. However, there was no significant difference in MHC I molecule expression between the cells exposed to isobutyl—DPD, n-hexyl—DPD and phenyl—DPD microparticles combined with the microparticulate gonorrhea vaccine in contrast to the cells exposed to only microparticulate gonorrhea vaccine (Figure 15A). This fact reveals that the ent—DPD and n-butyl—DPD microparticles have an adjuvant effect with the microparticulate gonorrhea vaccine, while isobutyl—DPD, n-hexyl—DPD, and phenyl—DPD microparticles do not.

We observed significantly higher expression of MHC II molecules from dendritic cells when exposed to the microparticulate formulations of ent—DPD, n-butyl—DPD, and isobutyl—DPD combined with the microparticulate gonorrhea vaccine in contrast to the cells exposed to only the microparticulate gonorrhea vaccine. However, no remarkable difference was observed in the expression of the antigen-presenting MHC II molecules between the cells exposed to the microparticulate formulations of n-hexyl—DPD and phenyl—DPD in combination with the gonorrhea vaccine microparticles compared to gonorrhea vaccine microparticles only (Figure 15B). Thus, microparticles of ent—DPD, n-butyl—DPD, and isobutyl—DPD were seen to show an adjuvant effect, while a similar adjuvant effect was not observed with n-hexyl—DPD and phenyl—DPD microparticles.

### 2.13. Evaluation of Adjuvant Effect: Expression of Costimulatory Molecules

**Figure 16:**
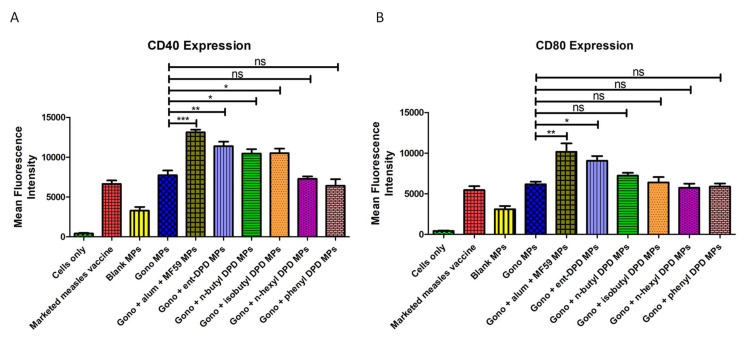
(**A**) CD40 expression; (**B**) CD80 expression on the surface of murine dendritic cells (DC 2.4). Results are expressed as the mean ± SEM (n = 3). * *p* < 0.05 significant; ** *p* < 0.01 very significant; *** *p* < 0.001 extremely significant.

The murine dendritic cells (DC 2.4) showed a significantly higher expression of CD40 upon exposure to the microparticulate formulations of the ent—DPD, n-butyl—DPD, and isobutyl—DPD microparticles integrated with the gonorrhea vaccine microparticles in contrast to the cells that were exposed to only gonorrhea vaccine microparticles. However, there was no significant difference in the expression of the costimulatory molecule CD40 on the surface of dendritic cells (DC 2.4) while exposed to only gonorrhea vaccine microparticles and combined with n-hexyl—DPD and phenyl—DPD microparticles (Figure 16A). Thus, microparticles of ent—DPD, n-butyl—DPD, and isobutyl—DPD showed an adjuvant effect, while a similar adjuvant effect was not observed with n-hexyl—DPD and phenyl—DPD microparticles.

The dendritic cells showed a significantly higher expression of the costimulatory molecule CD80 when exposed to the ent—DPD microparticles in combination with the gonorrhea vaccine microparticles. However, there was no significant difference in the expression of the costimulatory molecule CD80 on the surface of dendritic cells when exposed to the microparticulate formulations of n-butyl—DPD, isobutyl—DPD, n-hexyl—DPD, and phenyl—DPD in combination with the microparticulate gonorrhea vaccine compared to the cells exposed to the gonorrhea vaccine microparticles alone (Figure 16B). This shows that the ent—DPD microparticles have an adjuvant effect with gonorrhea vaccine microparticles, while the microparticulate formulations of n-butyl—DPD, isobutyl—DPD, n-hexyl—DPD, and phenyl—DPD do not.

## 3. Discussion

Bacteria use quorum sensing (QS), as a type of complex communication process, among themselves to control the population density. Through this process, bacteria act collectively as a group instead of as a single organism. The higher tolerance of bacteria to antibiotics is attributed to the fact that bacteria can regulate its behavior through a QS communication process towards virulence and biofilm formation. Thus, disrupting this QS process could lead to the improved control of bacterial infections [19]. Autoinducer-2, also known as a universal autoinducer, acts as a QS molecule that can act upon both types of bacteria, Gram-positive and Gram-negative. This molecule is derived from its precursor (S)-4,5-dihydroxy-2,3-pentanedione (DPD) molecule [20].

The AI-2 precursor (S)-DPD molecule can undergo a rapid interconversion to various linear and cyclic forms that are perceived by different types of bacteria, rendering QS targeting difficult. Thus, we previously tested the potential of a microparticulate formulation of the AI-2 precursor, (S)-DPD, as a potential vaccine adjuvant. Microparticulate (S)-DPD showed significant adjuvant activity with the microparticulate gonorrhea vaccine in in vitro studies [23]. Based on these results, we further explored the potential of microparticulate formulations of various synthetic analogs of (S)-DPD (as described in Figure 1) as vaccine adjuvants with the microparticulate gonorrhea vaccine.

PLGA was chosen as the polymer matrix for the formulation of the microparticles. PLGA is a biocompatible and biodegradable material that provides effective protection to the encapsulated material [43,44]. Equal amounts of blank microparticles were used as controls in all experiments. This ensured that the blank particles were nonimmunogenic, and, thus, the immunogenicity and adjuvanticity could be attributed to the analogs of (S)-DPD in the formulation [45]. In the studies, the negative control was dendritic cells. However, the positive control, for instance, in Figure 3, was the marketed measles vaccine. Although there was a significant difference between the cells only group and the blank MP, the objective here was to compare the marketed vaccine versus our experiential adjuvant compounds. Generally, the blank MPs were expected to show a higher response than the cells only group, since the blank MP group was in a microparticulate form. This is one of the advantages of a microparticulate formulation.

As per the World Health Organization (WHO) guidelines, the probable adjuvant candidate should first be tested for its cytotoxic effect [46]. Thus, we examined the cytotoxic effect of microparticulate formulations of various analogs of (S)-DPD toward dendritic cells using an MTT assay. We found that the microparticulate formulations of all the analogs of (S)-DPD tested were noncytotoxic toward dendritic cells. We performed a dose response for the microparticulate DPD analogs (ent—DPD, n-butyl—DPD, isobutyl—DPD, n-hexyl—DPD, and phenyl—DPD) in concentrations from 50 µg/mL (low) to 500 µg/mL (high). In the cytotoxicity study, the objective was to determine an appropriate dose in vitro that could be used for further studies. In Figure 4, it shows that at low doses, such as 50 µg/mL, the DC or cell viability was very high. However, as the concentration increased, the cytotoxicity slowly increased. In the later studies, only a low dose was used considering the results found in the cytotoxicity study. In the past, from our laboratory, we have numerous published articles that showed an immunogenicity and cytotoxicity correlation between in vitro and in vivo assay, which indicated that the assays are biologically viable [38,39,43].

We further evaluated these microparticles for their ability to induce the innate immune response via a Griess’s assay. In this study, the microparticulate formulations of ent—DPD, n-butyl—DPD, and isobutyl—DPD were identified to be immunogenic as they showed notable nitric oxide release levels from dendritic cells. In order to evaluate a formulation in vivo, it should be evaluated in vitro first. In an in vitro investigation, the immunostimulatory potential of these DPD analogs can be measured by measuring the NO release from dendritic cells. DC cells release NO and other cytokines after they are stimulated. NO is the first chemical that is released that we can measure in vitro that gives a great indication of what may happen in vivo. There are several studies that mention NO as an indicator for measuring an innate response. In the past from our laboratory, we have numerous published studies that showed an immunogenicity correlation between in vitro and in vivo assays [39,40,44]. However, the microparticles of n-hexyl—DPD and phenyl—DPD were nonimmunogenic. The expression of antigen-presenting and costimulatory molecules also showed similar results.

The adjuvant potential evaluation of the MP adjuvants of analogs ent—DPD, n-butyl—DPD, isobutyl—DPD, n-hexyl—DPD, and phenyl—DPD for their adjuvant potential by combining them with several bacterial and viral vaccines also showed that some of the analogs have potential as adjuvants. MenAfriVac (MAV), a meningococcal A conjugate vaccine, was combined with several analogs of DPD (Figure 10). When tested with APCs, several adjuvants, including ent—DPD, n-butyl—DPD, and isobutyl—DPD, displayed similar immunostimulatory potential as the MAV. However, n-hexyl—DPD and phenyl—DPD did not produce significant NO release from APCs compared to the MAV vaccine alone or the positive control, LPS. The results indicated that the ent—DPD, n-butyl—DPD, and isobutyl—DPD MP adjuvants can be added to formulate an adjuvanted MAV vaccine. The second bacterial vaccine we evaluated was the Hemophilus influenzae type B (HIB) conjugate vaccine (Figure 11), which was combined with several adjuvants. Interestingly, only ent—DPD produced a significant response. However, the analog molecules n-butyl—DPD, isobutyl—DPD, n-hexyl—DPD, n-hexyl—DPD, and phenyl—DPD did not produce significant differences in comparison to the HIB vaccine alone. These results indicate that these small molecule microparticles may not produce a robust humoral or cellular immune response when formulated with the HIB vaccine. When several adjuvants were combined with the measles vaccine (Figure 12), only ent—DPD displayed a robust NO release, indicating that ent—DPD does have adjuvant potential. The other adjuvants, with analogs molecules n-butyl—DPD, isobutyl—DPD, n-hexyl—DPD, n-hexyl—DPD, and phenyl—DPD, did not produce a significant difference in NO compared to the measles vaccine alone. The results indicate that the measles vaccine can be combined with the ent—DPD adjuvant to formulate a vaccine. These compounds were then combined with the influenza (S) vaccine (Figure 13). Interestingly, only ent—DPD produced significantly higher NO compared to the vaccine alone. There was no adjuvant potential when the influenza (S) vaccine was combined with analog molecules of n-butyl—DPD, isobutyl—DPD, n-hexyl—DPD, and phenyl—DPD. Our results indicate that ent—DPD, combined with the influenza (S) vaccine, may produce a robust immune response. We also evaluated the immunostimulatory potential of our experimental microparticulate compounds with the inactivated Zika vaccine (Figure 14). For the Zika vaccine combined with several DPD analogs, only ent—DPD showed adjuvant potential in comparison to the vaccine alone. However, when the Zika vaccine was combined with analog molecules of n-butyl—DPD, isobutyl—DPD, n-hexyl—DPD, and phenyl—DPD, no significant benefit was observed, indicating that the addition of these two adjuvants may not further stimulate a robust immune response. In this case, a vaccine for the Zika virus can be formulated with adjuvant ent—DPD. Taken together, only ent—DPD was able to produce a significant NO level with several bacterial and viral vaccines.

Additionally, the induction of the innate immune response was evaluated as a measure of the adjuvant potential of the various microparticulate DPD analogs through exposure of the antigen-presenting cells to a combination of the microparticulate gonorrhea vaccine developed previously in our laboratory with various microparticulate DPD analogs [27]. Microparticulate DPD analogs were weighed up to the microparticulate formulations of FDA-approved adjuvants, including alum and MF59^®^, in terms of their ability to act as an adjuvant. In this study, the microparticulate formulations of ent—DPD, n-butyl—DPD, and isobutyl—DPD were shown to display a remarkable adjuvant effect with the microparticulate gonorrhea vaccine. However, microparticulate formulations of n-hexyl—DPD and phenyl—DPD did not show an adjuvant effect. These results were found to be consistent with our results obtained for the immunogenicity assessment of the microparticulate formulations of (S)-DPD analogs. The expression of the antigen-presenting molecules MHC I and MHC II, as well as their corresponding costimulatory molecules CD80 and CD40, on the surface of dendritic cells upon exposure to the combination of microparticulate gonorrhea vaccine with various microparticulate DPD analogs further confirmed this effect. The microparticulate formulations of n-hexyl—DPD and phenyl—DPD did not show an adjuvant effect. However, the microparticles of ent DPD, n-butyl—DPD, and isobutyl—DPD did display an adjuvant effect, with ent—DPD microparticles showing the highest adjuvant effect with the microparticulate gonorrhea vaccine.

In our previous work, we assessed the bacterial QS biomolecule N-octanol-L-homoserine lactone, which is also known as autoinducer-1 (C8-HSL), as a potential vaccine adjuvant in vaccine formulations [43]. In that study, the immunostimulatory potential of C8-HSL microparticles was compared with the current marketed FDA-approved microparticulate formulations of adjuvants such as Alhydrogel^®^ (alum), AddaVax™ (MF59), and cytosine-phosphorothioate-guanine oligodeoxynucleotides (CpG). It was further determined that C8-HSL MP is immunostimulatory and produces an immune response similar to that of FDA-approved adjuvants (alum, MF59, and CpG). In addition, adjuvant efficacy was noted when the C8-HSL microparticle was integrated with several particulate vaccines, such as the Zika microparticle vaccine, measles microparticle vaccine, and marketed influenza vaccine [43].

The needs for future study include the investigation of adjuvant effects of microparticulate formulations of (S)-DPD analogs with an additional number of vaccines to confirm the adjuvanticity. Moreover, the concentration-dependent adjuvanticity of microparticulate formulations of (S)-DPD analogs and the in vivo study that will evaluate the other aspects of immunogenicity, such as antibody titers and cytokine level measurements that will provide clear insight into the safety, immunogenicity, and adjuvanticity of these formulations. At an early development stage, the microparticulate formulations of ent—DPD, n-butyl—DPD, and isobutyl—DPD were determined to be suitable adjuvant candidates regarding their formulation, non-cytotoxicity, and adjuvant effect.

## 4. Materials and Methods

### 4.1. Materials

The analogs of (S)-DPD were purchased from Omms Scientific Inc. (Dallas, TX, USA). Poly(vinyl alcohol) (PVA) and poly(lactic-co-glycolic acid) (PLGA) (50:50) were purchased from Sigma-Aldrich, St. Louis, MO, USA. Dendritic cells (DC 2.4) for in vitro study were obtained from ATCC. Adjuvants alum and AddaVax^TM^ were purchased from InvivoGen, San Diego, CA, USA. The measles vaccine was obtained from the Serum Institute of India Pvt. Ltd. Pune, India. Antibodies used to stain murine MHC I, MHC II, CD40, and CD80 molecules for flow cytometric analysis were purchased from eBioscience laboratories (San Diego, CA, USA). Dulbecco’s modified Eagle’s medium (DMEM), fetal bovine serum (FBS), penicillin/streptomycin, and nonessential amino acids were purchased from Cellgro Mediatech (Herndon, VA, USA).

### 4.2. Methods

#### 4.2.1. Formulation of Microparticles

**Figure 17:**
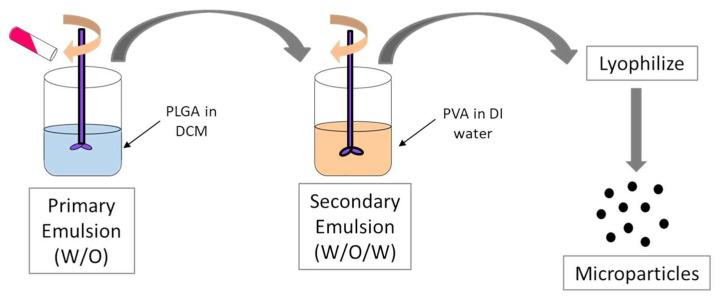
Schematic representation of the method for formulation of microparticulate DPD analogs.

Microparticles were formulated using the double-emulsion solvent evaporation method [44,47,48]. A 1% *w/v* solution of PLGA (50:50) was made by dissolving PLGA (50:50) in dichloromethane (DCM). Span 80 was added as an emulsifier. The aqueous solution of the various analogs of (S)-DPD (2% *w*/*w*) was then added and homogenized using the Omni TH_Q_ probe homogenizer (18,000 rpm) equipment to make the primary W/O emulsion. The emulsion was then ensued by adding with 0.1% *w/v* solution of PVA and homogenized using a probe homogenizer (18,000 rpm) to form the W/O/W double emulsion. The formulation was kept in an ice bath throughout the process. The emulsion was kept on a magnetic stirrer for 5 h to completely evaporate the dichloromethane (DCM). The double emulsion was then ultracentrifuged to concentrate the microparticles. Trehalose was then added as the cryoprotectant, and the emulsion was lyophilized to obtain the dried microparticles (Figure 17). Furthermore, all three FDA approved adjuvants, Alum, MF59, and CpG, were made into microparticles. These microparticles were formulated using the double-emulsion solvent evaporation method as described here.

#### 4.2.2. Microparticle Recovery Yield

The recovery yield was determined for all the microparticles formulated. The following formula was employed to calculate the percent yield of microparticles:

(1) $$percent\;recovery\;yield=\frac{Weight\;of\;microparticles\times 100}{Weight\;of\;all\;ingredients\;in\;the\;formulation}$$

#### 4.2.3. Particle Size and Zeta Potential

To determine the size and zeta potential of the particles, 5 µg of microparticles was suspended in deionized water (1 mL). Then, the size and surface zeta potential of the microparticles were measured in a Malvern Nano ZS [19,31,38]

#### 4.2.4. Scanning Electron Microscopy

The surface morphology of the microparticles loaded with various DPD analogs was visualized using scanning electron microscopy (SEM). Microparticles were dissolved in water and mounted using double-sided adhesive tape. Then, the surface morphology was evaluated using an SEM (scanning electron microscope) (Phenome benchtop SEM obtained from Nanoscience instruments, Phoenix, AZ, USA).

#### 4.2.5. Griess’s Assay for Nitrite

Nitric oxide release from the dendritic cells was measured to assess the immunogenicity of microparticles to trigger an immune response [24,49,50]. Griess’s assay was used to quantify nitrite, an oxidation product of nitric oxide, to evaluate the release of nitric oxide from dendritic cells. Various microparticulate formulations, including microparticles of ent DPD, n-butyl—DPD, isobutyl—DPD, n-hexyl—DPD, and phenyl—DPD, were incubated with murine dendritic cells (DC 2.4) (50 × 10^4^ cells/well) in 48-well plates for 48 h. Blank (unloaded) microparticles and marketed measles vaccine were used as negative and positive controls, respectively. The solutions were then transferred to another plate which was then added with Griess’s reagents of 1% sulfanilamide in 5% phosphoric acid and 0.1% NED (N-1-naphthylethylenediamine dihydrochloride in deionized water. This was then incubated in the dark for 10 min, which was followed by measuring the absorbance at 540 nm using a BioTek Synergy H1 plate reader (BioTek Instruments, Winooski, VT, USA). A standard curve was obtained by using a 100 µM solution of sodium nitrite to quantify the concentration of nitrite.

#### 4.2.6. Cytotoxicity Measurement

The MTT (3-(4,5-dimethylthiazol-2-yl)-2,5-diphenyltetrazolium bromide) assay was employed to analyze the toxicity of the microparticulate DPD analogs toward murine dendritic cells (DC 2.4) [42] DC 2.4 cells (2.5 × 10^5^ cells/well, in 96-well plate) were placed at various concentrations of microparticulate DPD analogs for 48 h at 37 °C. At the end of 48 h, MTT reagent (5 µg/mL in PBS) was added to the supernatants from all the wells and incubated at 37 °C for 4 h, protected from light. The precipitate formazan was then dissolved by the addition of DMSO (dimethyl sulfoxide) and shaking the plate for 15 min at room temperature, protected from light. At the end of 15 min, the absorbance was quantified using a BioTek Synergy H1 plate reader (BIO-TEK Instruments, Winooski, VT, USA) at 570 nm.

#### 4.2.7. In Vitro Release Study

An in vitro release study of C8-HSL was conducted to determine the percentage of DPD analogs released from the PLGA polymer [43]. Briefly, 5 mg of each analog of DPD (ent—DPD, n-butyl—DPD, isobutyl—DPD, n-hexyl—DPD, phenyl—DPD) MP was weighed in a balance, which was then added to Eppendorf tubes, where each tube contained 1 mL of phosphate buffered saline (PBS). Each of the Eppendorf tubes was then placed in an incubator at 60 rpm at 37 °C temperature. The supernatants were collected and replaced with PBS at 0, 1, 2, 3, to 168 h. The sample was then centrifuged at 1500 rpm for 10 min. After completing the centrifuge, the small molecule content that was released was determined via a spectrophotometer (UV) at different wavelengths for the following analogs: ent—DPD (230 nm), n-butyl—DPD (234 nm), isobutyl—DPD (240 nm), n-hexyl—DPD (242 nm), and phenyl—DPD (245 nm) (Thermo-Fisher, Waltham, MA, USA, NANODROP 2000c).

#### 4.2.8. Expression of Antigen-Presenting Molecules

Dendritic cells (DC 2.4) were exposed to different microparticulate formulations. The level of major histocompatibility complexes I and II was quantified. Dendritic cells in population concentration of 50 × 10^4^ cells/well in a 48-well plate were exposed to different groups such as marketed measles vaccine (positive control), blank microparticles, ent DPD microparticles, n-butyl—DPD microparticles, isobutyl—DPD microparticles, n-hexyl—DPD microparticles, and phenyl—DPD microparticles groups which was then (n = 3) incubated at 37 °C for 48 h. After completion of incubation, the cells were stained with allophycocyanin (APC1) and fluorescein isothiocyanate-labeled MHC I and MHC II markers (eBioscience Laboratories, San Diego, CA, USA) for 1 h at 4 °C, protected from light. The fluorescence intensity was quantified using a BD Accuri C6 plus flow cytometer (BD Bioscience, San Jose, CA, USA).

#### 4.2.9. Expression of Costimulatory Molecules

Antigen-presenting dendritic cells (DC 2.4) were exposed to the different groups of DPD–analogs microparticulate formulations. The level of CD40 and CD80 (costimulatory molecules) was quantified. Dendritic cells with population density of 50 × 10^4^ cells/well in a 48-well plate were exposed to different groups, including marketed measles vaccine (positive control), blank microparticles, ent—DPD microparticles, n-butyl—DPD microparticles, isobutyl—DPD microparticles, n-hexyl—DPD microparticles, and phenyl—DPD microparticles (n = 3) groups, which were then incubated at 37 °C for 48 h. After completion of incubation, the cells were stained with allophycocyanin (APC1) and fluorescein isothiocyanate-labeled CD40 and CD80 markers (eBioscience Laboratories, San Diego, CA, USA) for 1 h at 4 °C, protected from light. The fluorescence intensity was measured using a BD Accuri C6 plus flow cytometer (BD Bioscience, San Jose, CA, USA).

#### 4.2.10. Griess’s Assay

The efficacy of microparticles as adjuvants was analyzed by quantifying the nitric oxide released from dendritic cells when exposed to different DPD analog microparticulate formulations integrated with gonorrhea vaccine microparticles [27]. Nitrite, an oxidation product of nitric oxide, was quantified to evaluate the release of nitric oxide from dendritic cells (DC 2.4). Dendritic 2.4 cells with populations of 50 × 10^4^ cells/well in a 48-well plate were exposed to different DPD analog groups, including alum + MF59^®^ microparticles, ent—DPD microparticles, n-butyl—DPD microparticles, isobutyl—DPD microparticles, n-hexyl—DPD microparticles, and phenyl—DPD microparticles, combined with gonorrhea vaccine microparticles for 48 h. The concentration of nitrite was then quantified as described in Section 4.2.5.

#### 4.2.11. Adjuvant Effect: Expression of Antigen-Presenting Molecules

Various microparticulate DPD analogs were combined with a microparticulate gonorrhea vaccine and exposed to dendritic cells (DC 2.4) to evaluate the adjuvant effect by assessing the expression of major histocompatibility complex I and II, antigen-presenting molecules. DC 2.4 cells (50 × 10^4^ cells/well in a 48-well plate) were exposed to the combination of various microparticulate DPD analogs and microparticulate formulations of FDA-approved adjuvants (alum and AddaVax^TM^) with gonorrhea vaccine microparticles (n = 3), which were then incubated at 37 °C temperature for 48 h. Major histocompatibility complex I and II expression on the surface of the antigen-presenting dendritic cells (DC 2.4) was then quantified as described in Section 4.2.8..

#### 4.2.12. Adjuvant Effect: Expression of Costimulatory Molecules

Various microparticulate DPD analogs were combined with a microparticulate gonorrhea vaccine and exposed to dendritic cells (DC 2.4) to evaluate the adjuvant effect by assessing the expression of CD40 and CD80, costimulatory molecules. DC 2.4 cells (50 × 10^4^ cells/well in a 48-well plate) were exposed to the combination of various microparticulate DPD analogs and microparticulate formulations of FDA-approved adjuvants (alum and AddaVax^TM^) with gonorrhea vaccine microparticles (n = 3), which were then incubated at 37 °C temperature for 48 h. The expression of costimulatory molecules CD40 and CD80 on the surface of the antigen-presenting dendritic cells (DC 2.4) was then evaluated as described in Section 4.2.9.

#### 4.2.13. Statistical Analysis

All experiments were replicated three times. GraphPad Prism 5 software was used for statistical analyses. The results are represented as the mean ± SEM. An unpaired two-tailed *t* test was used for comparison of different experimental groups. A one-way ANOVA followed by Tukey’s post hoc analysis was used for comparison of multiple groups. In this study, all the experiments were conducted in triplicate unless otherwise stated. A Shapiro–Wilk test was used to test the normality of the data and the Brown Forsythe test was used to evaluate the equality of variances. A one-way analysis of variance (ANOVA) with Tukey’s post hoc test was performed for datasets with normal distribution.

## 5. Conclusions

This study screened five DPD analogs, ent—DPD((R)-4,5-dihydroxy-2,3-pentanedione), n-butyl—DPD ((S)-1,2-dihydroxy-3,4-octanedione), isobutyl—DPD ((S)-1,2-dihydroxy-6-methyl-3,4-heptanedione), n-hexyl—DPD ((S)-1,2-dihydroxy-3,4-decanedione), and phenyl—DPD ((S)-3,4-dihydroxy-1-phenyl-1,2-butanedione) of (S)-4,5-dihydroxy-2,3-pentanedione, for use as vaccine adjuvants. The microparticulate formulations of these analogs of (S)-DPD were found to be noncytotoxic toward dendritic cells. The microparticulate formulations of ent DPD, n-butyl—DPD, and isobutyl—DPD have the potential to serve as probable vaccine adjuvant candidates with further research in the field.

## Figures and Tables

**Table 1:** Advantages and limitations of current vaccine technologies.

Vaccine Technologies	Advantages	Limitations
Live-attenuated	▪ Produces a robust humoral and cellular responses	▪ Production is time consuming
Inactivated	▪ Original pathogen used to stimulate immune responses ▪ Can produce quickly	▪ Immunogenicity may dwindle with increasing newer strains
Subunit	▪ Safe ▪ Easy to manufacture	▪ Lower immunogenicity ▪ May need adjuvant in formulation
Viral Vector	▪ Produces robust humoral and cellular responses	▪ Immunogenicity and safety concerns
Nucleic acid DNA	▪ Higher safety profile ▪ Easy to manufacture	▪ Lower transfection efficiency ▪ Lower protein expression
Messenger RNA (mRNA)	▪ Quick preparation ▪ Produces robust humoral and cellular responses	▪ Can cause side effects

**Table 2:** Formulation and characterization of microparticulate DPD analogs. Values in parentheses were measured before lyophilization.

Parameter	Mean ± SEM				
	ent DPD	n-butyl—DPD	Isobutyl—DPD	n-hexyl—DPD	Phenyl—DPD
Recovery Yield (%)	90.12 ± 2.1	87.87 ± 2.3	89.94 ± 1.7	90.61 ± 2.4	91.49 ± 1.9
Particle size (nm)	462.85 ± 5.85 (351.63 ± 4.49)	368.2 ± 10.46 (273.34 ± 6.17)	380.2 ± 16.26 (247.38 ± 13.11)	357.47 ± 4.99 (286.46 ± 5.16)	468.26 ± 16.41 (402.61 ± 14.69)
Polydispersity index (PDI)	0.74 (0.52)	0.49 (0.31)	0.71 (0.63)	0.82 (0.68)	0.83 (0.62)
Zeta potential (mV)	−33.3 ± 7.12 (−31.7 ± 4.63)	−29.9 ± 8.22 (−30.1 ± 7.39)	−30.6 ± 6.57 (−28.5 ± 3.61)	−32.9 ± 4.78 (−30.7 ± 6.14)	−35.0 ± 7.76 (−32.8 ± 4.37)

## Data Availability

The data presented in this study are available on request from the corresponding author.
